# Elevated expression and altered processing of fibulin-1 protein in human breast cancer

**DOI:** 10.1038/sj.bjc.6600802

**Published:** 2003-03-18

**Authors:** L M Greene, W O Twal, M J Duffy, E W McDermott, A D Hill, N J O'Higgins, A H McCann, P A Dervan, W S Argraves, W M Gallagher

**Affiliations:** 1 Department of Pharmacology, Conway Institute of Biomolecular and Biomedical Research, University College Dublin, Belfield, Dublin 4, Ireland; 2 Department of Cell Biology and Anatomy, Medical University of South Carolina, 173 Ashley Avenue, Charleston, SC, USA; 3 Department of Nuclear Medicine, St Vincent's University Hospital, Elm Park, Dublin 4, Ireland; 4 Department of Surgery, St Vincent's University Hospital, Elm Park, Dublin 4, Ireland; 5 Department of Pathology, Conway Institute of Biomolecular and Biomedical Research, University College Dublin, Belfield, Dublin 4, Ireland; 6 Mater Hospital, Eccles Street, Dublin 7, Ireland

**Keywords:** fibulin, breast cancer, extracellular matrix, proteolysis, oestrogen receptor

## Abstract

The extracellular matrix protein fibulin-1 suppresses the motility and invasiveness of a variety of tumour cell types *in vitro* as well as the growth of fibrosarcoma tumours in nude mice. In this study, fibulin-1 protein expression in breast carcinoma specimens and normal breast tissue was evaluated immunohistologically. Fibulin-1 protein expression was also semiquantitatively assessed by immunoblot analysis in a collection of normal breast tissues (*n*=18), benign tumours (*n*=5) and breast carcinomas (*n*=39). In normal breast tissue, fibulin-1 protein expression predominated in the ductal epithelium and underlying myoepithelium, with weaker staining evident in the loose connective surrounding the ducts. Examination of breast carcinomas revealed that the tumour cells also expressed fibulin-1 protein. The level of mature fibulin-1 polypeptide (100 kDa) was higher in the breast carcinoma specimens as compared to normal breast tissue (Mann–Whitney *U*-test, *P*=0.0005). In addition to the mature fibulin-1 polypeptide, several smaller sized polypeptides of 55, 50 and 25 kDa were detected using monoclonal antibodies reactive towards an epitope located at the N-terminus of fibulin-1. The immunoreactive 50 kDa polypeptide was detected more frequently in breast carcinoma specimens than in normal breast tissue (*χ*^2^=17.22, *P*<0.0001). Furthermore, the ratio of the 50 kDa fragment to the mature fibulin-1 polypeptide correlated with the level of oestrogen receptor *α* (Spearman correlation coefficient, rs=0.49, *P*<0.003, *n*=36) and progesterone receptor (rs=0.43, *P*=0.008, *n*=36) expression in the tumour specimens. Taken together, these findings indicate that elevated expression and altered processing of fibulin-1 is associated with human breast cancer.

Fibulin-1 is an extracellular matrix and plasma protein that has been implicated as playing a role in tumour progression (Qing *et al*, 1997; Hayashido *et al*, 1998; Twal *et al*, 2001). Alternative splicing of fibulin-1 precursor results in four transcript variants, designated A–D, of which C and D are predominantly expressed (Tran *et al*, 1997). Overexpression of fibulin-1D in fibrosarcoma-derived cells has been shown to delay tumour formation in nude mice and suppress invasion into gels of reconstituted basement membrane extracts (Qing *et al*, 1997). Furthermore, human placenta-derived fibulin-1 protein has also been shown to inhibit cell adhesion, spreading and motility of a range of human tumour cell lines, including those originating from melanoma, epidermoid carcinoma and breast carcinoma (Twal *et al*, 2001). A recent study showed increased fibulin-1C : 1D mRNA ratios in ovarian carcinomas (Moll *et al*, 2002). Additionally, fibulin-1C expression is induced by oestradiol in oestrogen receptor *α* (ER*α*)-positive ovarian tumour cells (Clinton *et al*, 1996; Moll *et al*, 2002). These results suggest that the biological function of fibulin-1 during tumour progression may be variant-specific.

Despite there being substantial evidence for fibulin-1 having the ability to influence tumour cell behaviour, there is a very limited understanding of the expression of fibulin-1 protein in association with human cancers. Indeed, there are only two reports to date (both from the same research group) that document the expression of fibulin-1 in human cancer, specifically ovarian carcinoma (Roger *et al*, 1998; Moll *et al*, 2002). In this study, we evaluated fibulin-1 protein expression in human breast tumours. We also sought to correlate fibulin-1 expression levels with the oestrogen receptor status of the breast tumour specimens.

## MATERIALS AND METHODS

### Handling of tumours

The breast tissue samples used in this study were obtained from the Department of Surgery, St Vincent's University Hospital, Dublin and the Department of Pathology, Mater Private Hospital, Dublin. Patient characteristics, including histological details and steroid receptor status, are summarised in Table 1

**Table 1:** Patient and histological data associated with tumor specimens

**Characteristic**	***n* (%)**
*Patients*	
*Total* n	62 (100)
Normals	18 (29)
Benign tumours	
Fibroadenomas	4 (6.5)
Phylloides	1 (1.6)
Breast Carcinomas	
Primary tumours	36 (58.1)
Lymph node metatastasis	3 (4.8)
	
*Carcinomas*	
*Size* (cm)	
⩽2	6 (15.4)
>2	30 (76.9)
Unknown	3 (7.7)
*Histological type*	
Ductal	27 (69.2)
Lobular	7 (18)
Unknown	5 (12.8)
*Grade*	
1	2 (5.1)
2	10 (25.6)
3	20 (51.3)
Unknown	7 (18)
*Nodal status*	
Negative	7 (18)
Positive	27 (69.2)
Unknown	5 (12.8)
*ERα status*^a^	
Positive	20 (51.3)
Negative	16 (41)
Unknown	3 (7.7)
*PR status*^b^	
Positive	15 (38.5)
Negative	21 (53.8)
Unknown	3 (7.7)

a ER*α*=oestrogen receptor alpha.

b PR=progesterone receptor.

. ER*α* and progesterone receptor (PR) status of the breast tumours was determined by cytosolic assays (Cullen *et al*, 2001). The cutoff point for ER*α* and PR status was 200 fmol g^−1^. Normal breast tissue specimens were obtained from sites either remote from the primary tumour or from cosmetic reduction mammoplasties.

### Immunohistochemistry

Tumour specimens were fixed in 3.7% paraformaldehyde for 24 h, embedded in paraffin wax and sectioned at 5 *μ*m thickness. Sections were deparaffinised with xylene and graded ethanol, followed by rehydration with phosphate-buffered saline (PBS) at pH 7.3. Endogenous peroxidase activity was quenched by incubating the sections with 3% hydrogen peroxide in PBS for 10 min. Sections were subsequently washed with PBS and blocked by incubation with 5% normal horse serum in PBS (blocking solution A) for 20 min at room temperature (RT). Sections were then incubated with the mouse antifibulin-1 monoclonal antibody (mAb) 3A11, diluted 1/500 (3 *μ*g ml^−1^) in blocking solution A, for 2 h at RT. After washing with PBS, immunoglobulin G (IgG) detection was performed with the Vectastain Elite ABC kit (Vector Laboratories, Peterborough, UK) according to the manufacturer's recommendations. Staining was then visualised using 0.05% 3,3-diaminobenzidine/0.01% hydrogen peroxide (Vector Laboratories). Nuclei were counterstained using methyl green (Vector Laboratories). Sections were then dehydrated and mounted in distrene dibutylpthalate xylene. Immunostaining specificity was checked using an irrelevant mouse mAb (UPC10; Sigma, Dorset, UK) of the same immunoglobulin subclass (IgG2a) as the antifibulin-1 mAb.

### Immunoblot analysis

Breast tissue specimens were rapidly frozen in liquid nitrogen and stored at −80°C. Tissue homogenisation was carried out using a Braun Micro Dismembrator (Braun, Melsungen, Germany. Twenty-five milligram of tissue powder was extracted by a brief vortex in 250 *μ*l RIPA buffer (50 mM Tris, 150 mM NaCl pH 7.4, 1% NP-40, 0.5% deoxycholate, 0.1% sodium dodecyl sulphate (SDS), 10 mM EDTA, 2 mM phenylmethylsulphonyl fluoride (PMSF) and 5 mM *N*-ethylmaleimide (NEM)) followed by incubation at 4°C for 1 h on a rotisserie. All extracts were then centrifuged at 13 000 **g** for 15 min. Supernatants were removed and the protein content of the extracts determined using the bicinchoninic acid protein assay kit (Pierce Chemical, Rockford, IL, USA) with bovine serum albumin as the protein standard. Protein extracts were stored in aliquots at −80°C.

Forty microgram of protein was mixed with an equal volume of SDS sample buffer, separated on a 10% SDS/polyacrylamide gel and subsequently electroblotted onto a polyvinylidene difluoride (PVDF) membrane (Bio-Rad, Hercules, CA, USA). Electroblotted proteins were visualised via Ponceau Red staining. Human fibulin-1 protein, isolated from human placenta by immunoaffinity chromatography (Balbona *et al*, 1992), was used as a positive control for immunoblotting. Electroblotted membranes were blocked for 2 h at RT in Tris-buffered saline (TBS) (50 mM Tris, 150 mM NaCl pH 7.6) containing 0.1% (v v^−1^) Tween-20 and 5% (w v^−1^) nonfat dried milk (blocking solution B). The membranes were washed briefly in TBS/0.1% Tween-20 and then probed overnight at 4°C with mouse antifibulin-1 mAb 3A11 diluted 1/1500 (1 *μ*g ml^−1^) in 5% blocking solution B. Following 3 × 10 min washes in TBS/0.1% Tween-20, membranes were incubated with horseradish peroxidase-conjugated goat anti-mouse IgG (Promega, Madison, WI, USA) diluted 1/2500 in 5% blocking solution for 30 min at RT. After washing, immunoreactive complexes were detected by enhanced chemiluminescence using ECL Plus (Amersham Pharmacia Biotech, England) and recorded by exposure to BioMax ML autoradiographic film (Eastman Kodak Company, Rochester, NY, USA).

The PR status of the human breast cancer cell lines was confirmed by immunoblot analysis. Probed membranes were stripped and reprobed overnight at 4°C with 1 *μ*g ml^−1^ mouse anti-PR mAb (clone PR4-12) (Oncogene Research Products, Cambridge, UK). Following detection, blots were again stripped and reprobed for 1.5 h at RT with the panspecific mouse antiactin mAb (clone JLA20) (Oncogene), diluted 1/5000 in 5% blocking solution B, to control for equal loading of protein. Bound antibodies were detected as above.

### Cells

Three ER/PR-positive human breast cancer cell lines (MCF-7, T-47-D and BT-474) and three ER/PR-negative human breast cancer cell lines (MDA-MB-231, HS-578-T and SK-BR-3) were used in this study. All cell lines were obtained from the European Collection of Cell Cultures (http://www.ecacc.org/). MCF-7 cells were grown in minimal essential medium (MEM) (Sigma, Dorset, UK) supplemented with 1% nonessential amino acids (Sigma). T-47-D and MDA-MB-231 cells were grown in Dulbecco's modified Eagle's medium (DMEM) (GIBCO BRL, Life Technologies, UK). HS-578-T cells were grown in DMEM supplemented with 10 *μ*g ml^−1^ insulin (Sigma). BT-474 cells were grown in RPMI-1640 (Sigma). SK-BR-3 cells were grown in McCoy's 5a medium modified (Sigma). All the above-mentioned cell culture medium types were supplemented with 10% foetal calf serum (FCS), 2 mM L-glutamine, penicillin (100 units ml^−1^) and streptomycin (100 *μ*g ml^−1^) (GIBCO). Cells were maintained at 37°C in 5% CO_2_ at 100% humidity. At 5 to 7 day intervals, cells were passaged using trypsin–EDTA (GIBCO).

### Effect of oestrogen on fibulin-1 processing by cultured cells

Steroids were depleted from heat-inactivated FCS with dextran-coated-activated charcoal (DCC) (Sigma) (Devleeschouwer *et al*, 1987). Cells were grown for 5 days in phenol red-free medium containing 10% DCC-treated FCS. Cells were then washed with phenol red-free medium and treated for 48 h with either 10 nM oestradiol, or ethanol vehicle in phenol red-free medium minus serum. After 48 h, the conditioned culture medium (CCM) was removed and both PMSF and NEM added to 2 and 5 mM final concentration, respectively. Cell debris was removed by centrifugation. The supernatant was then concentrated using Centricon YM-30 filters (Millipore, Malborough, MA, USA) for 1 h at 3000 **g**. The protein content of the concentrated CCM was determined as described above. The remaining cell layer was washed twice with ice-cold PBS and extracted using RIPA buffer supplemented with 10 mM EDTA. The extract was then centrifuged at 13 000 **g** for 15 min at 4°C. The supernatant was removed and protein concentration of the extract determined. Forty microgram aliquots of protein were evaluated by immunoblot analysis as described above.

### Quantification and statistical analysis

Radiographs from immunoblot experiments were scanned and densitometric analysis performed using a GDS 8000 system, together with Gel Works 1D analysis software (Ultra-Violet Products Ltd, Cambridge, England). Nonspecific background was subtracted and band intensity values derived. Comparisons between unpaired groups were performed using the nonparametric Mann–Whitney *U*-test. Associations between either fibulin-1 expression or processing and clinicopathological parameters were examined using the Spearman rank correlation test or with dichotomised variables using the *χ*^2^ test of independence. All reported *P*-values refer to a two-sided test of significance. *P*-values ⩽0.05 were considered to be significant.

## RESULTS

### Fibulin-1 protein expression in human breast cancers and normal breast tissue

**Figure 1:**
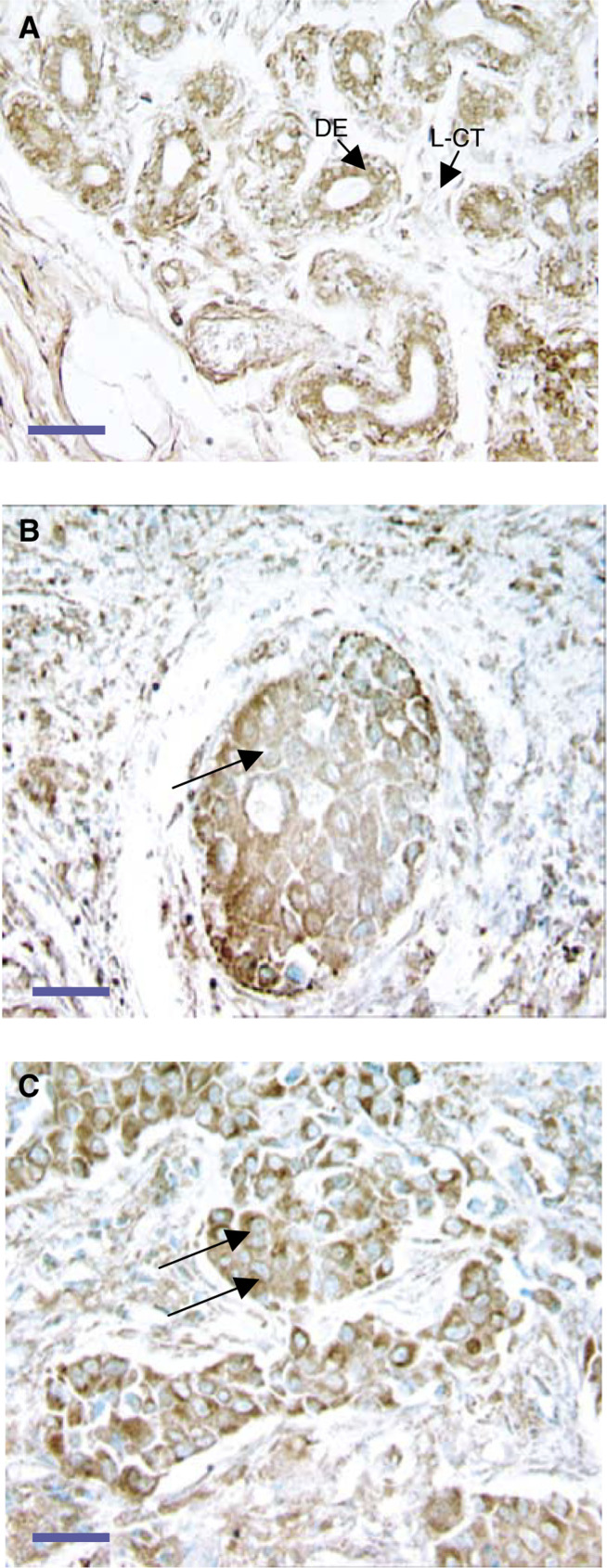
Fibulin-1 protein expression in normal breast tissue and invasive ductal carcinoma. Paraffin-embedded tissue sections of normal breast tissue (**A**), ductal carcinoma *in situ* (**B**) and invasive ductal carcinoma (**C**) were stained with antifibulin-1 3A11 mAb (brown). In (**A**), immunostaining is prominent in the epithelial layer of the duct (DE), with weaker staining apparent within the L-CT surrounding the ducts. In (**B**), the carcinoma cells of solid-type ductal carcinoma *in situ* stained markedly with antifibulin-1 antibody (arrow). In (**C**), fibulin-1 protein expression is apparent in the invasive ductal carcinoma cells (extended arrows). Sections were counterstained with methyl green. Bars indicate 20 *μ*m. Immunostaining specificity was checked using an irrelevant mouse mAb of the same IgG subclass (data not shown).

Fibulin-1 protein expression was examined immunohistochemically in human breast tissue using a mouse anti-human fibulin-1 mAb (clone 3A11). The 3A11 antibody reacts with an epitope located at the N-terminus of fibulin-1 and detects all four documented splice variants of the protein (Argraves *et al*, 1990). In normal breast tissue, fibulin-1 protein expression predominated in the ductal epithelium and underlying myoepithelium, with weaker staining evident in the loose connective tissue (L-CT) surrounding the ducts (Figure 1A

). Examination of ductal carcinoma *in situ* (Figure 1B) and invasive breast carcinomas (Figure 1C) revealed that tumour cells expressed fibulin-1 protein, with the connective tissue surrounding these tumour cells also exhibiting moderate expression of fibulin-1.

**Figure 2:**
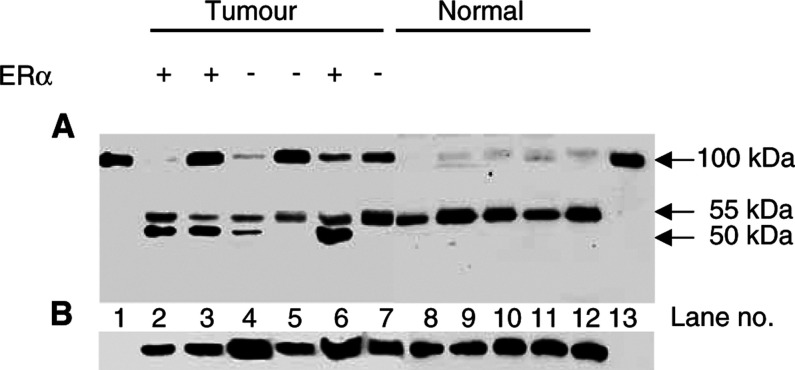
Analysis of fibulin-1 protein expression in representative breast tissue extracts. In (**A**), purified human fibulin-1 protein (lanes 1 and 13) and 40 *μ*g of breast tumour extracts (lanes 2–7) and 40 *μ*g of normal breast tissue (lanes 8–12) extract. Samples were run on a 10% polyacrylamide gel under reducing conditions, transferred to PVDF membrane and probed with the 3A11 mAb, which is directed against the N-terminus of human fibulin-1. In addition to the mature fibulin-1 polypeptide (apparent molecular mass of 100 kDa) immunoreactive fragments of 55 and 50 kDa are detectable. The ER status of the breast tumours is also indicated. In (**B**), the blot shown in (**A**) was stripped and reprobed with a panspecific antiactin antibody to serve as a control for protein loading.

Fibulin-1 protein expression was semiquantitatively assessed in a range of breast carcinoma and normal breast tissue biopsies by densitometric analysis of immunoblots. Purified fibulin-1 protein, derived from human placenta, was used as a positive control. Under reducing conditions, the 3A11 mAb specifically recognised the 100 kDa placenta-derived fibulin-1 protein (Figure 2A

, lanes 1 and 13). In detergent extracts from breast tissue (Figure 2A, lanes 2–12), three major immunoreactive bands were detected, one of which corresponds to the 100 kDa mature fibulin-1 polypeptide, with two additional bands at 55 and 50 kDa. These smaller fragments represent processed forms of fibulin-1, most likely generated by proteolysis of the full-length fibulin-1 polypeptide.

**Figure 3:**
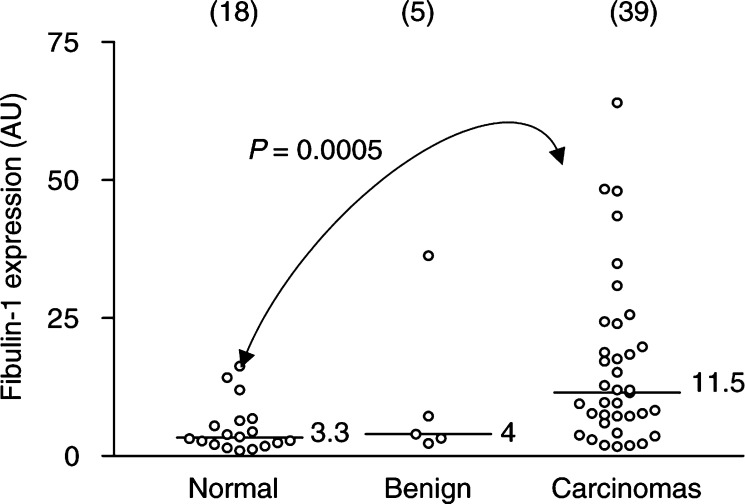
Comparison of full-length fibulin-1 protein expression in normal breast tissues (*n*=18), benign tumours (*n*=5) and breast carcinomas (*n*=39). Levels of 100 kDa fibulin-1 were quantified by densitometric analysis and expressed in arbitrary units. Bar, median value. *P*-values were determined according to the nonparametric Mann–Whitney *U*-test for unpaired values. Full-length fibulin-1 protein was significantly elevated in the breast carcinoma specimens as compared to normal breast (*P*=0.0005).

A comparison of 100 kDa fibulin-1 expression in normal breast tissue, benign tumours and breast carcinomas is shown in Figure 3

. Fibulin-1 protein expression was significantly increased in breast carcinomas as compared to normal breast tissue (Mann–Whitney *U*-test, *P*=0.0005). An increase in fibulin-1 protein expression was also observed in the breast carcinomas compared to benign tumours, but this difference did not reach significance (Mann–Whitney *U*-test, *P*=0.2), probably owing to the low number of benign tumours examined. Regarding 100 kDa fibulin-1 expression, no significant difference was observed between normal breast tissue and benign tumours (Mann–Whitney *U*-test, *P*=0.3). The 55 kDa immunoreactive fragment was equally detected in both normal and carcinoma breast tissue (Figure 2). However, the 50 kDa fragment was found more frequently in benign breast tumours (five out of five, 100%) (*χ*^2^=4.55, *P*=0.03) and in breast carcinomas (35 out of 39, 90%) (*χ*^2^=17.22, *P*<0.0001) than in normal breast tissue (six out of 18, 33%). In summary, these results provide evidence for elevated expression and altered processing of fibulin-1 protein in breast tumour development and progression.

### Association of fibulin-1 expression with prognostic variables

Full-length fibulin-1 protein is known to be secreted by oestrogen-responsive ovarian cancer cells upon exposure to oestradiol (Clinton *et al*, 1996). In addition, fibulin-1 expression was previously shown to be inversely correlated with PR protein levels in ovarian carcinomas (Roger *et al*, 1998). However, expression of 100 kDa fibulin-1 in the breast carcinomas examined here did not correlate with either ER*α* or PR status (Table 2

**Table 2:** Relationship between prognostic variables and fibulin-1 expression and processing

**Clinical characteristics**	***n*^a^**	**Fibulin-1 expression^b^**	***P*^c^**	**Fibulin-1 processing^d^**	***P*^e^**
*Tumour size*					
⩽2 cm	6	15		19.9	
>2 cm	30	19.2	0.4	18.2	0.7
Unknown	3				
					
*Histological type*					
Ductal	27	16.5		17.6	
Lobular	7	21.3	0.3	17.1	0.9
Unknown	5				
					
*Histological grading*					
G1,G2	12	16.8		13.3	
G3	20	16.3	0.9	18.4	0.1
Unknown	7				
					
*Nodal status*					
Negative	7	19.3		12.7	
Positive	20	16.4	0.5	18.7	0.2
Unknown	12				
					
*ERα status*^f^					
Positive	20	15.9		21.7	
Negative	16	21.8	0.09	14.6	0.045
Unknown	3				
					
*PR status*^g^					
Positive	15	16.9		21.6	
Negative	21	19.6	0.4	16.6	0.2
Unknown	3				

a *n*, number of breast carcinomas including 36 primary breast carcinomas and three lymph node metastases

b Level of full-length (100 kDa) fibulin-1 polypeptide as determined by densitometric analyses of immunoblots (represented in arbitrary units).

c ,

e *P*-values were calculated using a nonparametric Mann–Whitney *U*-test for comparison of means.

d Fibulin-1 processing was determined as the ratio of the 50 kDa fragment to the mature form (i.e. 50/100 kDa).

f ER*α*=oestrogen receptor alpha.

g PR=progesterone receptor.

). Previous studies have suggested benefits in viewing ER*α* and PR levels as continuous rather than dichotomous variables (Chapman *et al*, 1996). Therefore, we examined the relation of fibulin-1 expression with ER*α* and PR protein concentrations using the Spearman rank correlation test. Accordingly, fibulin-1 protein expression was inversely correlated with ER*α* protein concentration (rs=−0.34, *P*<0.04, *n*=36), but not with PR levels (rs=−0.18, *P*=0.3, *n*=36) (Figure 5A and B

**Figure 5:**
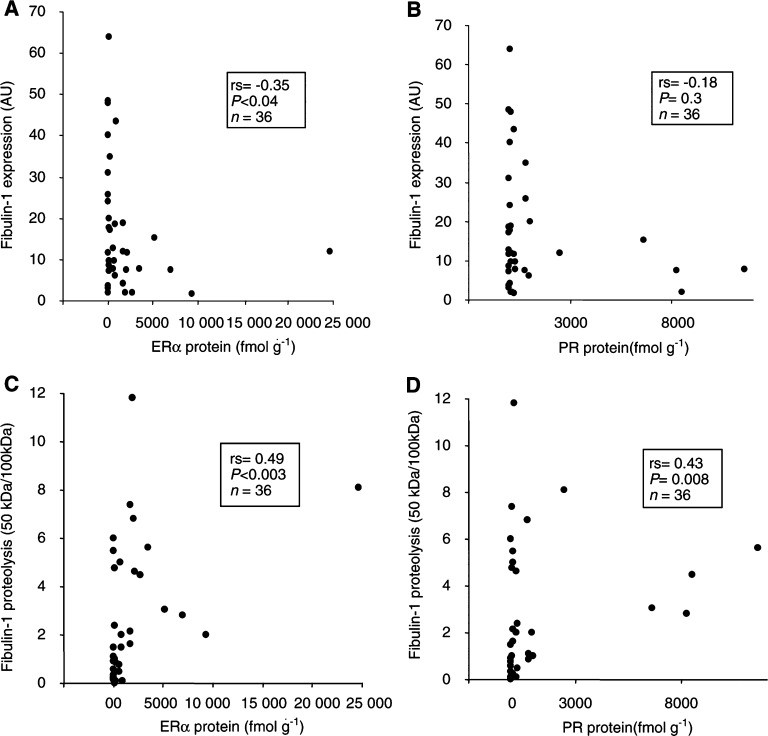
Relationship between fibulin-1 expression (**A**, **B**) or processing (**C**, **D**) and ER*α* (**A**, **C**) and PR (**B**, **D**) protein concentration. Fibulin-1 expression and processing was determined as in Figures 3 and 4

**Figure 4:**
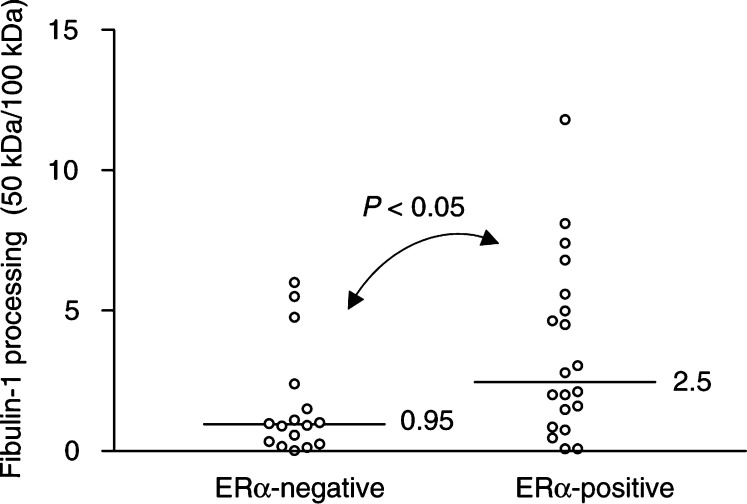
Relationship between fibulin-1 processing and ER status. Levels of 100 kDa fibulin-1 and the 50 kDa processed fragment were quantified by densitometric analysis of immunoblots. The resulting arbitrary units were used to determine the extent of fibulin-1 processing (expressed as the ratio of the 50 kDa fragment to the full-length polypeptide). Bar, median value. *P*-values were determined according to the nonparametric Mann–Whitney *U*-test for unpaired values. The extent of fibulin-1 processing (50 /100 kDa) was significantly higher in ER*α*-positive than in ER*α*-negative breast carcinomas (*P*<0.05).

, respectively. The ER*α* and PR concentrations were determined by cytosolic assays. There was a significant inverse correlation between fibulin-1 expression and ER*α* protein concentration (rs=−0.34, *P*<0.04, *n*=36; the Spearman rank correlation). In addition, the correlation between fibulin-1 processing and ER*α* (rs=0.49, *P*<0.003) and PR receptor (rs=0.43, *P*<0.008) status was highly significant.

).

When viewed as the ratio of the 50 kDa fragment to the mature fibulin-1 polypeptide, fibulin-1 processing was more extensive in ER*α*-positive as compared to ER*α*-negative breast carcinomas (*P* < 0.05, *n*=36) (Figures 2 and 4). A positive correlation was also found between fibulin-1 processing and absolute ER*α* protein concentration (rs=0.49, *P*<0.003, *n*=36) (Figure 5C). A similar correlation with fibulin-1 processing was observed with PR protein concentration (rs=0.43, *P*=0.008, *n*=36) (Figure 5D). As shown in Table 2, fibulin-1 protein expression and processing did not correlate with any other prognostic variables, including tumour size, histological type, tumour stage or lymph node involvement.

### Fibulin-1 processing in human breast tumour-derived cells

**Figure 6:**
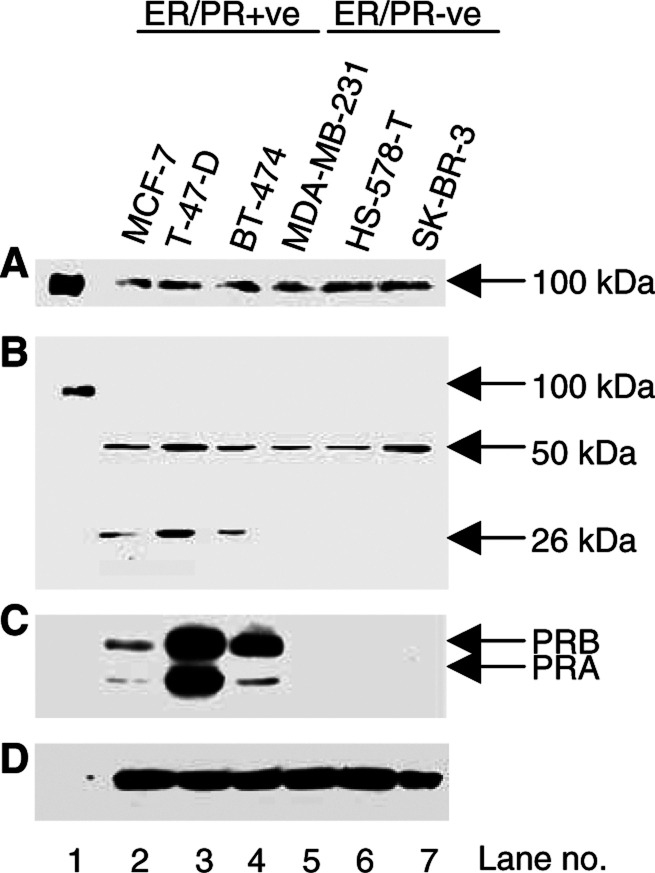
Fibulin-1 expression and processing in ER/PR-positive and ER/PR-negative human breast tumour-derived cell lines. In (**A**) and (**B**), aliquots of concentrated CCM and detergent extracts of the cells, respectively, were immunoblotted using antifibulin-1 mAb. In (**C**), blot *B* was stripped and reprobed with an anti-PR mAb that detects two isoforms of the PR protein, PRB (120 kDa) and PRA (94 kDa). Only the ER/PR-positive cell lines expressed detectable levels of PR protein. In (**D**), blot *B* was again stripped and reprobed with the antiactin mAb.

As processing was associated with both ER*α* and PR in human breast carcinomas, we next studied the relation of fibulin-1 processing *in vitro* using three ER/PR-positive (MCF-7, T47D and BT-474) and three ER/PR-negative (MDA-MB-231, HS-578-T and SK-BR-3) human breast tumour-derived cell lines. Immunoblot analysis of fibulin-1 was performed on both cell extracts and CCM (Figure 6

). Full-length fibulin-1 protein was detected in the CCM (Figure 6A), but not in the cell extract (Figure 6B) of the six human breast tumour-derived cell lines analysed. The 50 kDa fragment was detected at different levels in the various cell extracts examined, with lower amounts being expressed in two ER/PR-negative breast cancer cell lines (MDA-MB-231 and HS-578-T). An additional 26 kDa fragment was detected in the three ER/PR-positive cell lines (Figure 6B). The 26 kDa fragment was also detected in two out of 36 (5.5%) of the breast carcinomas (data not shown). Notably, this subset of carcinomas expressed strikingly high levels of both ER*α* and PR. The 50 kDa fragment may represent an intermediate product, which can be further processed into a 26 kDa fragment. Alternatively, the 26 kDa fragment may be directly derived from the mature 100 kDa fibulin-1 polypeptide via degradation. The absence of the 55 kDa fragment in any of the breast tumour cell lines examined may be reflective of a stromal origin for this fragment in breast tissue. Overall, fibulin-1 processing was more extensive in the ER/PR-positive as compared to ER/PR-negative breast tumour cell lines.

### Fibulin-1 processing is not regulated by oestrogen in T47D breast tumour cells

**Figure 7:**
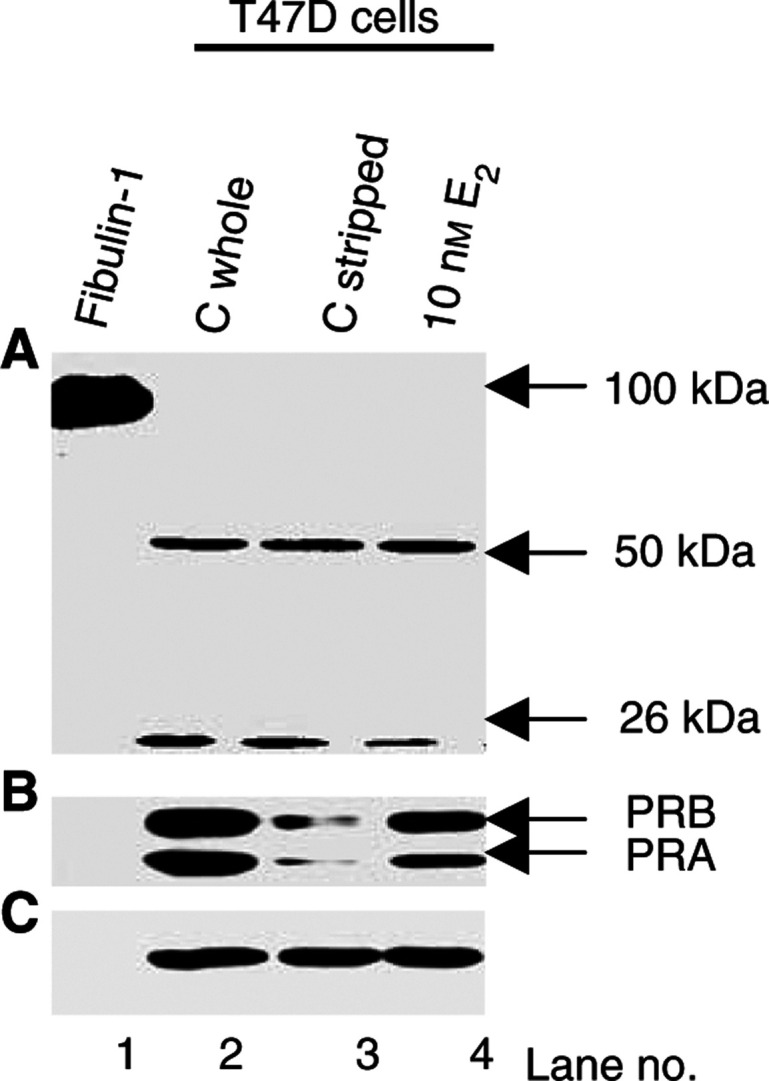
Western blot analysis of the effect of oestradiol (E_2_) on fibulin-1 processing. Purified fibulin-1 protein (lane 1) and aliquots of detergent extracts from T47D cells grown in either endogenous steroid-containing serum-free medium (lane 2; C whole), steroid-depleted serum-free medium containing ethanol vehicle (lane 3; C stripped) or steroid-depleted serum-free medium containing 10 nM exogenous E_2_ (lane 4) were immunoblotted using antifibulin-1 3A11 mAb (**A**), anti-PR mAb (**B**), and antiactin mAb (**C**). See Materials and Methods for details of culture conditions. Blots are representative of three independent experiments.

**Figure 8:**
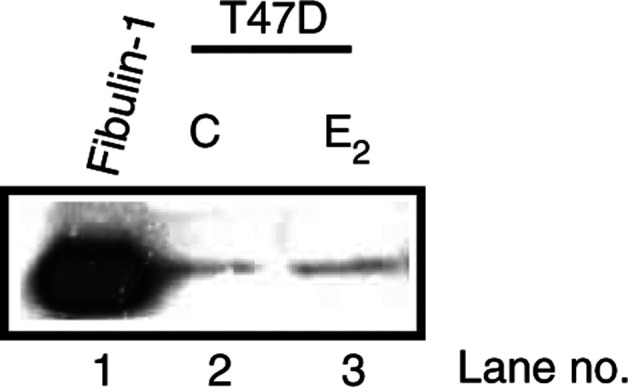
Western blot analysis of the effect of oestradiol (E_2_) on fibulin-1 expression. Purified fibulin-1 protein (lane 1) and aliquots of concentrated CCM (10 ml original volume) from either steroid-depleted serum-free medium containing ethanol vehicle (lane 2) or steroid-depleted serum-free medium containing 10 nM exogenous E_2_ (lane 3) were immunoblotted using antifibulin-1 3A11 mAb. See Materials and Methods for details of culture conditions. Blot is representative of three independent experiments.

We then examined the effect of oestrogen on fibulin-1 processing in the ER/PR-positive T47D cell line. Cells were deprived of steroids by culturing for 5 days in 10% DCC-FCS phenol red-free DMEM. Both the rate of cell proliferation and levels of oestrogen-inducible proteins, such as the PR, are significantly reduced when the cells are cultured in serum pretreated with dextran charcoal (Hurd *et al*, 1999; Iwasaki *et al*, 1999). The charcoal treatment is 98% effective in removing oestradiol (Devleeschouwer *et al*, 1987). As shown in Figure 7B

, the level of PR is significantly reduced when the cells are grown for 5 days in stripped serum (lane 3; C stripped) compared with cells grown in control whole serum (lane 2; C whole). Exposure of the cells to 10 nM oestradiol for 48 h reverses this effect (lane 4), indicating reactivation of the ER pathway. However, alteration in ER function via either steroid depletion or re-exposure to oestrogen did not affect fibulin-1 processing (Figure 7A). Similarly, the level of fibulin-1 secreted into the medium from T47D cells was not altered in response to oestrogen stimulation (Figure 8

).

## DISCUSSION

The present investigation demonstrates that elevated fibulin-1 protein expression is associated with breast cancer. This work is in agreement with a recent study showing increased expression of fibulin-1 mRNA in a small panel of breast carcinoma specimens as compared to normal breast tissue (Forti *et al*, 2002). In our study, fibulin-1 protein was detected via immunhistochemistry in both the stromal and epithelial compartments of normal and neoplastic breast tissue. In addition, we observed no significant alteration in the expression of the 55 kDa processed form of fibulin-1 between normal- and tumour-derived breast tissue. Thus, it is unlikely that the increase in the expression of full-length fibulin-1 protein in breast carcinoma is due primarily to an increase in the ratio of epithelial cells. This issue may be resolved in future studies examining the expression of fibulin-1 in specific cellular compartments isolated by microdissection. In the case of ovarian cancer, while fibulin-1 mRNA is produced by tumour-derived epithelial cells, fibulin-1 protein has been shown to accumulate in stroma surrounding the tumour cells (Roger *et al*, 1998). Given that the same mAb was used in both studies, it may be concluded that fibulin-1 protein accumulates in different compartments in a context-specific manner.

A number of other ECM proteins display modulated expression in association with cancer. For example, fibronectin and tenascin are upregulated in breast cancer (Mackie *et al*, 1987; Loridon-Rosa *et al*, 1988; Koukoulis *et al*, 1993; Ishihara *et al*, 1995; Takei *et al*, 1995,^1998^; Jahkola *et al*, 1996), while laminin-5 expression is increased in gliomas, gastic carcinomas, and squamous carcinomas, but downregulated in prostate and breast carcinomas (Martin *et al*, 1988). At least one common denominator between these proteins is that they each influence cell adhesion and migration (Akiyama *et al*, 1995; Giese *et al*, 1996; Romberger, 1997; Greenwood and Murphy-Ullrich, 1998; Hirosaki *et al*, 2000; Twal *et al*, 2001). Fibulin-1 has been shown to suppress the adhesion and motility of a number of tumour cell lines, including the breast carcinoma cell line MDA-MB-231. The elevated expression of fibulin-1 protein seen in breast and ovarian carcinomas (Clinton *et al*, 1996) may be reflective of a similar role *in vivo*. The recent observation that ovarian carcinomas exhibit an increased ratio of fibulin-1C : 1D mRNA (Moll *et al*, 2002) suggests that these fibulin-1 variants exhibit differing functions. It is tempting to propose that the balance of fibulin-1 variant expression is an important determinant of whether the expressed fibulin-1 displays tumour-suppressive or oncogenic activities.

In addition to elevated expression of the full-length (100 kDa) fibulin-1 polypeptide in breast carcinomas *vs* normal breast tissue, we also observed differential production of additional fibulin-1 immunoreactive fragments. The exact origin of these N-terminal fragments is still unclear, but the most likely mechanism of generation is via proteolysis of the mature fibulin-1 polypeptide. Indeed, fibulin-1 proteolysis has already been described *in vitro* (Sasaki *et al*, 1996) and in studies of cultured skin fibroblastic cells (Debeer *et al*, 2002), as well as in the skin of mice exhibiting 2,4-dinitrofluorobenzene-induced chronic contact dermatitis (Kusubata *et al*, 1999). Other explanations for the origin of the N-terminal fibulin-1 fragments include differential alternative splicing and/or the usage of internal ribosome entry sites.

In this study, we indicate that altered fibulin-1 processing is associated with breast cancer. Specifically, a 50 kDa N-terminal fragment of fibulin-1 was detected more frequently in breast carcinomas as compared to normal breast tissue. Moreover, an increased ratio of the 50 kDa fragment to the mature fibulin-1 polypeptide was observed in ER*α*-positive breast carcinomas as compared to ER*α*-negative carcinomas. Assuming that the observed immunoreactive polypeptides correspond to proteolytic fragments of fibulin-1, our results indicate that altered proteolysis of fibulin-1 is also associated with breast cancer. The 55 kDa fragment of fibulin-1 detected in both normal and breast cancer tissues may represent a precursor form, which is further digested to the 50 kDa fragment. On the other hand, it may represent a phosphorylated version of the 50 kDa fragment.

Additionally, the 26 kDa fragment was only detectable in ER*α*-positive breast carcinomas and ER*α*-positive breast cancerderived cell lines. Digestion of mouse fibulin-1 protein with human leukocyte elastase produces an N-terminal fragment of approximately 26 kDa (Sasaki *et al*, 1996), which may correspond to the immunoreactive fragment observed in this study. Intriguingly, elastases are also elevated in breast cancer (Kao and Stern, 1986). Furthermore, proteinase-like peptidase activities were previously found to be higher in patients with steroid receptor-rich breast tumours than in receptor-poor tumours (Vasishta *et al*, 1989). If the observed fragments are derived by proteolysis of mature fibulin-1 polypeptide, it is reasonable to hypothesise that the altered processing of fibulin-1 is reflective of differential proteinase activity in the breast tumours.

Fibulin-1C mRNA expression can be induced by oestrogen in ER*α*-positive ovarian tumour cell lines (Clinton *et al*, 1996); however, its association with ER*α* status in ovarian cancers is still unclear (Clinton *et al*, 1996; Moll *et al*, 2002). Interestingly, we found an inverse relation between the levels of full-length fibulin-1 protein and ER*α* protein concentration in breast cancers. We also found no evidence for augmented secretion of fibulin-1 by ER*α*-positive T47D breast cancer cells treated with oestrogen. While fibulin-1C and -1D variants differ at the C-terminus, both yield similar sized mature polypeptides as determined by immunoblot analysis. Indeed, currently available antibodies are still not capable of determining which fibulin-1 variant is predominantly expressed. It remains to be seen as to whether hormonal regulation of fibulin-1 expression is variant-specific.

In conclusion, our results have shown that fibulin-1 protein is elevated in human breast carcinomas as compared to normal breast tissue. Upregulation of fibulin-1 protein may be useful as a marker for breast and ovarian cancer. Clearly, additional investigations are required to determine if enhanced fibulin-1 protein expression is associated with other tumour types and if such altered expression is variant-specific. Also, further analysis will be required to determine if the processed fragments of fibulin-1 retain biological activity.
